# Genome-wide analysis of differentially expressed lncRNAs and mRNAs in primary gonadotrophin adenomas by RNA-seq

**DOI:** 10.18632/oncotarget.13948

**Published:** 2016-12-15

**Authors:** Jiye Li, Chuzhong Li, Jianpeng Wang, Guidong Song, Zheng Zhao, Haoyuan Wang, Wen Wang, Hailong Li, Zhenye Li, Yazhou Miao, Guilin Li, Yazhuo Zhang

**Affiliations:** ^1^ Beijing Neurosurgical Institute, Capital Medical University, Tiantan Xili, Dongcheng District, Beijing, China; ^2^ Beijing Neurosurgical Institute, Beijing Tiantan Hospital, Beijing Institute for Brain Disorders Brain Tumor Center, China National Clinical Research Center for Neurological Diseases, Capital Medical University, Beijing, China; ^3^ Department of Neurosurgery, The Affiliated Hospital of Qingdao University, Qingdao, Shandong Province, China; ^4^ Department of Neurosurgery, Zhujiang Hospital, Southern Medical University, Guangzhou, China; ^5^ Department of Neurosurgery, Beijing Tiantan Hospital, Capital Medical University, Beijing, China; ^6^ Department of Neurosurgery, Navy General Hospital, Beijing, China

**Keywords:** gonadotrophin adenoma, transcriptomics, ncRNA, pathway analysis, co-expression network

## Abstract

Recently, long non-coding RNAs (lncRNAs) have received increased research interest owing to their participation via distinct mechanisms in the biological processes of nonfunctional pituitary adenomas. However, changes in the expression of lncRNAs in gonadotrophin adenoma, which is the most common nonfunctional pituitary adenomas, have not yet been reported. In this study, we performed a genome-wide analysis of lncRNAs and mRNAs obtained from gonadotrophin adenoma patients’ samples and normal pituitary tissues using RNA-seq. The differentially expressed lncRNAs and mRNAs were identified using fold-change filtering. We identified 839 lncRNAs and 1015 mRNAs as differentially expressed. Gene Ontology analysis indicated that the biological functions of differentially expressed mRNAs were related to transcription regulator activity and basic metabolic processes. Ingenuity Pathway Analysis was performed to identify 64 canonical pathways that were significantly enriched in the tumor samples. Furthermore, to investigate the potential regulatory roles of the differentially expressed lncRNAs on the mRNAs, we constructed general co-expression networks for 100 coding and 577 non-coding genes that showed significantly correlated expression patterns in tumor cohort. In particular, we built a special sub-network of co-expression involving 186 lncRNAs interacting with 15 key coding genes of the mTOR pathway, which might promote the pathogenesis of gonadotrophin tumor. This is the first study to explore the patterns of genome-wide lncRNAs expression and co-expression with mRNAs, which might contribute to the molecular pathogenesis of gonadotrophin adenoma.

## INTRODUCTION

Pituitary adenomas account for 10–15% of intracranial neoplasms, which arise from adenohypophyseal cells [1, 2]. Nonfunctional pituitary adenomas (NFPAs) are the most common pituitary macroadenomas, comprising more than 30% of all pituitary tumors [3]. In addition to a very rare fraction of functioning gonadotrophin adenomas (GA), GA is generally considered “silent”or“nonfunctioning” and comprises 29%–35% of NFPAs. The tumor often causes neurological symptoms, including erectile dysfunction, headache, visual field defects, and hypopituitarism. There are no approved medical therapies for these tumors, and they typically require surgical intervention; however, surgery usually fails to cure GA because of its invasiveness or recurrence [4]. X-chromosomal inactivation analysis showed that the majority of pituitary adenomas are monoclonal [5]. A previous study showed that adenoma formation was probably caused by the clonal expansion of a single cell resulting from an intrinsic somatic pituitary cell genetic alteration [6]. Currently, the mechanism and epigenetic features of GA remain unknown. The identification of more precise and novel biomarkers to predict GA progression and invasion is imperative.

Among transcripts, about 10–20% are protein-coding RNAs, and the remaining 80%–90% are non-protein-coding RNAs. Long non-coding RNAs (lncRNAs) are non-encoding RNAs that are longer than 200 nucleotides, with little or no protein-coding capacity [7, 8]. The development of lncRNA high-throughput sequencing and bioinformatics technology have led to the discovery of lncRNAs’ participation in a wide range of biological processes, such as nuclear-cytoplasmic trafficking, dosage compensation, immune responses, cell growth, apoptosis, migration, and invasion, despite their lack of protein coding capability [9, 10]. Recently, increasing evidence has shown that lncRNAs play crucial and complex roles in tumor development and progression, including bladder cancer, gastric cancer, glioblastoma, and other types of malignant tumors [11–13]. Research has demonstrated that the expression of the tumor suppressor lncRNA maternally expressed gene 3 (MEG3) is selectively lost in NFPAs and that MEG3 can suppress tumor growth through activation of the p53 pathway [14, 15]. Specifically, overexpression of lncRNA Hox transcript antisense intergenic RNA (HOTAIR) has been demonstrated in NFPA, and is higher in invasive NFPA compared to in non-invasive NFPA [16]. However, the mechanistic properties of these dysregulation lncRNAs in GA remain obscure. In addition, integrated analysis correlating changes in the expression patterns of lncRNAs and mRNAs might be beneficial to predict their functional role in the pathogenesis and development of GA. As far as we know, there has been no study of the functional roles of the co-expression of lncRNAs and mRNA in GA. In this study, we identified differentially expressed lncRNAs and mRNA obtained from GA patients’ samples and normal pituitary tissues at the genome-wide level. We explored the genes targeted and regulated by the lncRNAs using gene ontology (GO) and Ingenuity Pathway Analysis (IPA). Finally, we constructed special co-expression networks to investigate mRNA–lncRNA interaction patterns via pathway analysis. The findings of this study provide a new insight into the pathogenesis and identification of novel biomarkers related to GA.

## RESULTS

### High throughput transcriptome sequencing identifies differentially expressed mRNAs and lncRNAs

**Figure 1:**
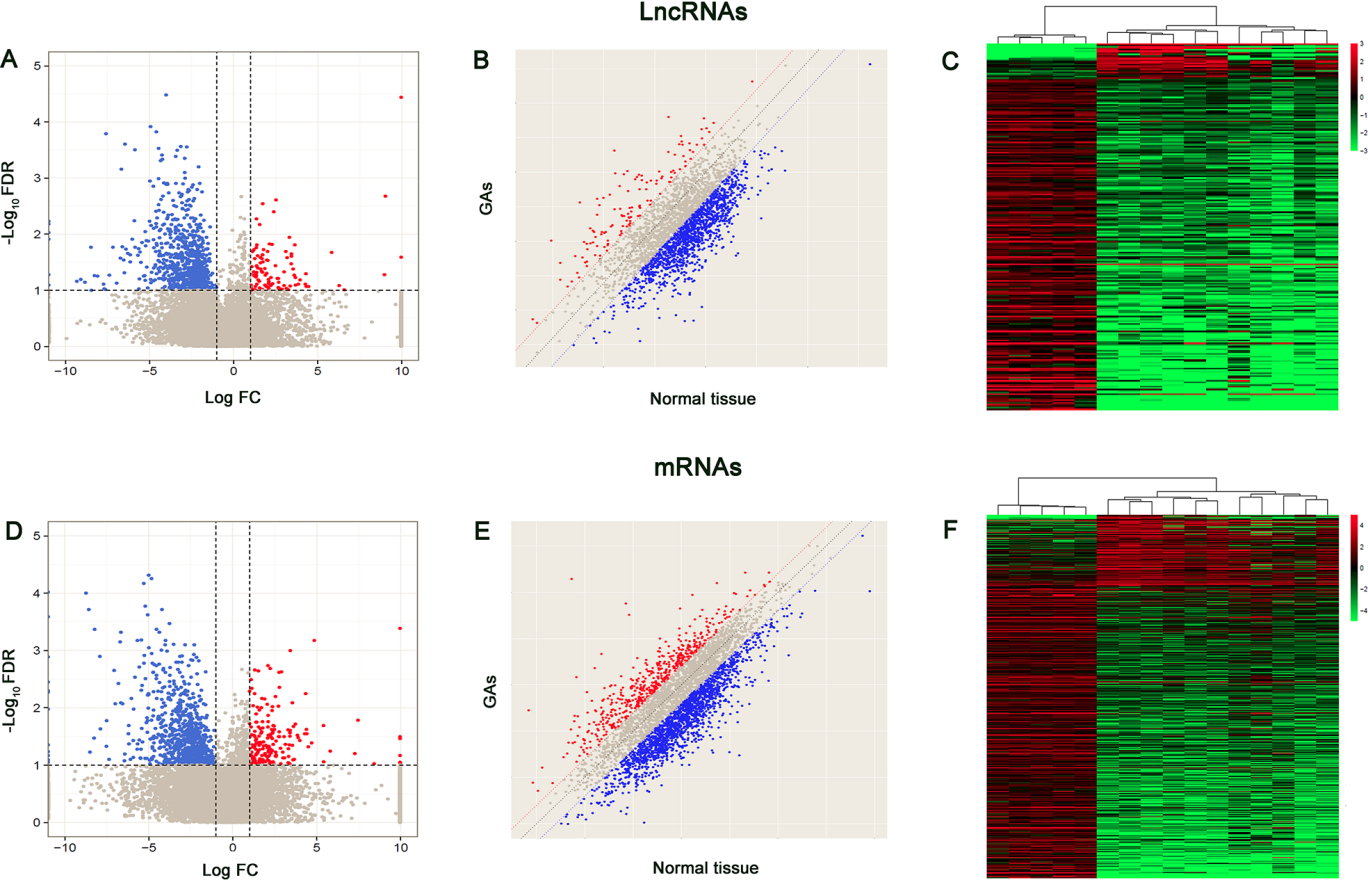
Volcano plots, scatter plots and heat map showing expression profiles of lncRNAs and mRNAs (**A**, **D**) Volcano plots. The negative log of FDR (base 10) is plotted on the Y-axis, and the log of the FC (base 2) is plotted on the X-axis. The red points on the graph A or D represent upregulated differently expressed lncRNAs and mRNAs in GA compared with normal tissue, respectively (FC > 2 and FDR< 0.1). The blue points represent downregulated lncRNAs and mRNAs (FC > 2 and FDR < 0.1) and the gray points represent LncRNAs and mRNAs with FC > 2 and FDR > 0.1. (**B**, **E**) The scatter-plot is used for evaluating the lncRNAs and mRNAs expression variation between GA samples and normal tissue samples. The values of x- and y-axes are averaged normalized values for each group (log2 scaled). The dashed lines represent fold change lines (the value of default fold change given is 2.0). The lncRNAs and mRNAs above the top red line and below the bottom blue line indicate a > 2-fold change of lncRNA and mRNA. (**C**, **F**) Differentially expressed lncRNAs and mRNAs (FC > 2 and FDR < 0.1) in GA and normal tissues are analyzed using hierarchical clustering. Each row represents a single LncRNA or mRNA and each column represents one sample. Red indicates high relative expression and green indicates low relative expression.

To investigate the possible biological functions of lncRNAs in GA, we analyzed lncRNA and mRNA expression profile data from GA samples and normal pituitary tissues using high throughput sequence technology. Genes with a fold-change (FC) > 2 or < 0.5 (for up- and downregulation, respectively), a *P* < 0.05, and a false discovery rate (FDR) < 0.1 were identified as significantly differentially expressed. As shown in Figure 1A and 1B, RNA-seq analysis identified 839 lncRNAs as significantly differentially expressed in tumor tissues, including 101 upregulated and 738 downregulated lncRNAs. Meanwhile, 200 and 815 mRNAs were upregulated and downregulated in GA samples compared to in controls, respectively (Figure 1D and 1E). Among these significantly differentially expressed LncRNAs, 243 displayed a fold-change > 10, including 22 upregulated lncRNAs and 221 downregulated lncRNAs. The most dysregulated lncRNAs and mRNAs are shown in Table 1. LINC00657 (fold change: > 500) was the most upregulated lncRNA. KCNQ1OT1 was one of the most upregulated lncRNAs in tumor issues compared to the control (fold change: ~57). An overview of the coding gene profile showed that 75 mRNAs had a fold-change > 10 (upregulated: 36; downregulated: 266). Hierarchical clustering showed that the expression patterns of the lncRNAs and mRNAs among the samples were obviously distinguishable (Figure 1C and 1F).

**Table 1:** The most dysregulated lncRNAs and mRNAs

Gene symbol	LncRNAs	Status	Gene symbol	mRNAs	Status
	Log_2_FC			Log_2_FC	
RP3-404K8.2	−12.5289	down	POMC	−14.4189	down
AC106870.2	−10.6285	down	GH1	−13.8967	down
LINC00657	9.9658	up	GH2	−13.0740	down
RP1-309H15.2	−9.3248	down	PRL	−12.2965	down
AC073284.4	−9.0888	down	FGFRL1	−11.8589	down
RP11-582J16.5	9.0239	up	ARHGEF28	−10.9256	down
TTN-AS1	8.966	up	DFNA5	−10.2130	down
AC133785.1	−8.8453	down	CCDC144A	−10.1246	down
RP11-598C10.1	−8.4886	down	MGAT2	9.9658	up
RP11-532N4.2	−8.2682	down	H1FX	9.9658	up
RP11-554D14.7	−8.0883	down	SF3B5	9.9658	up
AC012307.2	−7.7326	down	BTG2	9.9658	up
RP11-398J5.1	−7.6951	down	PPIG	9.9658	up
RP11-166D19.1	6.6439	up	SLC6A15	−8.7415	down
AC005550.4	6.5819	up	GAL	−8.5745	down
RP11-246K15.1	6.2735	up	ALPK2	8.4247	up
KCNQ1OT1	5.8239	up	SCGB1A1	7.4549	up
RP11-545D19.1	4.454	up	CXCL13	7.2726	up
RP11-403I13.4	4.3186	up	ZNF804A	5.8067	up
IFNG-AS1	4.2951	up	ZBTB41	5.4197	up

**Figure 2:**
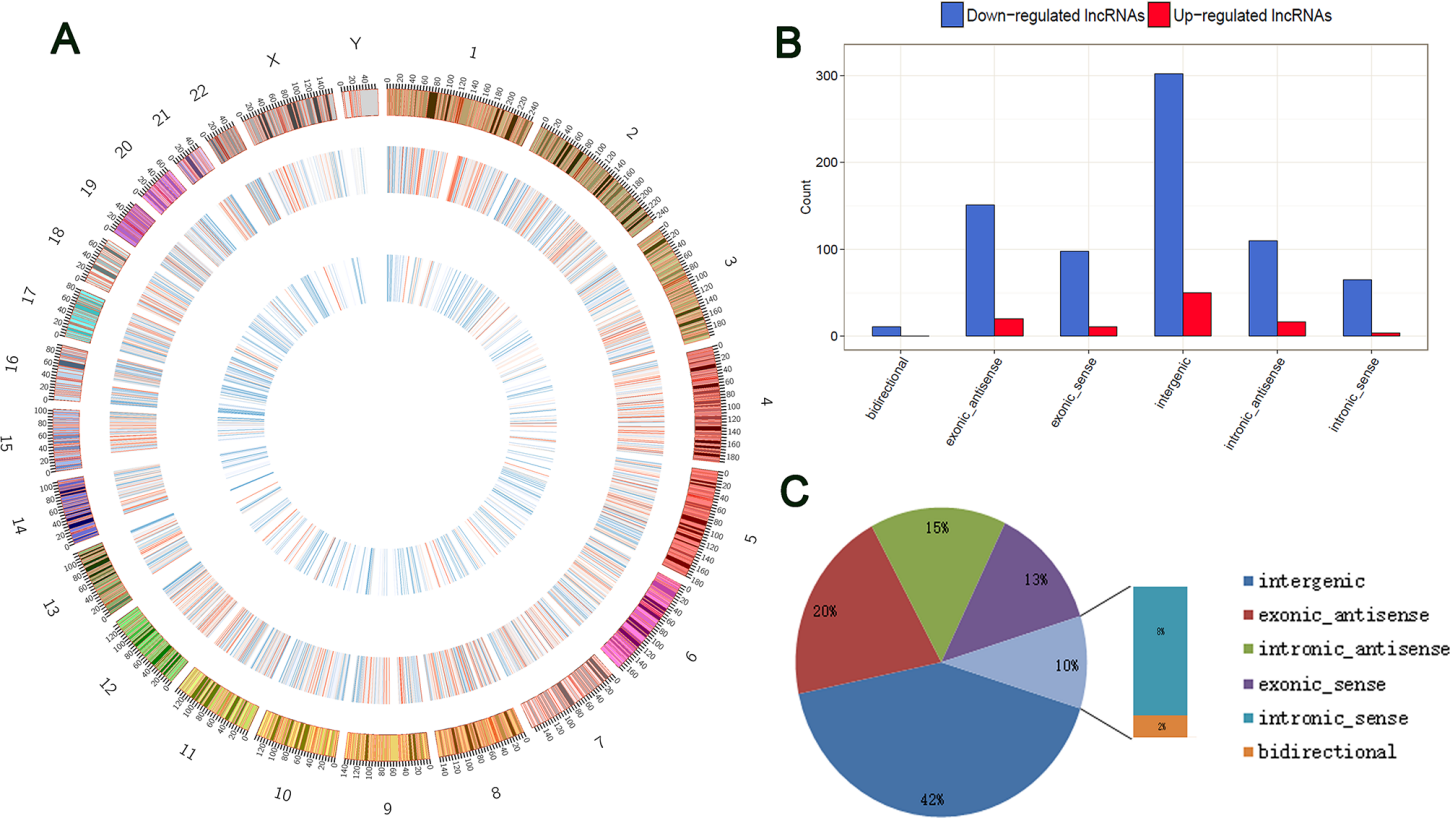
Identification of differentially expressed lncRNAs in GA (**A**) Circos plot showing lncRNAs on human chromosomes. The outermost layer of circos plot is chromosome map of the human genome. The increased or decreased lncRNAs have been marked in red or blue bars, respectively. The larger inner circle represents all target lncRNAs detected by RNA-seq, and the smaller inner circle indicates the significantly differentially expressed lncRNAs with fold change > 2.0 and FDR < 0.1. (**B**) Categories and counts of differentially expressed lncRNAs (FC > 2 and FDR < 0.1). The lncRNAs are classified into six types according to the relationship with their associated coding genes. (**C**) Pie charts show the proportion of six types of dysregulated LncRNAs in GA compared to the normal tissue.

These lncRNAs are widely distributed in all chromosomes, including the sex chromosomes (X and Y) (Figure 2A). According to their relation with protein-coding genes, the differentially expressed lncRNAs were classified into six categories: 12.9% were exonic sense-overlapping, 8.2% were intronic sense-overlapping, 20.3% were exonic antisense, 15.1% were intronic antisense, 42.0% were intergenic, and 1.3% were bidirectional (Figure 2B and 2C). Notably, intergenic lncRNAs accounted for the largest category (42.0%) among all differentially expressed lncRNAs, and we detected a greater proportion of intergenic lncRNAs among the total upregulated (5.8%) and downregulated lncRNAs (36.1%) in GA samples compared with those in the control.

### GO analysis of differentially expressed mRNAs

**Figure 3:**
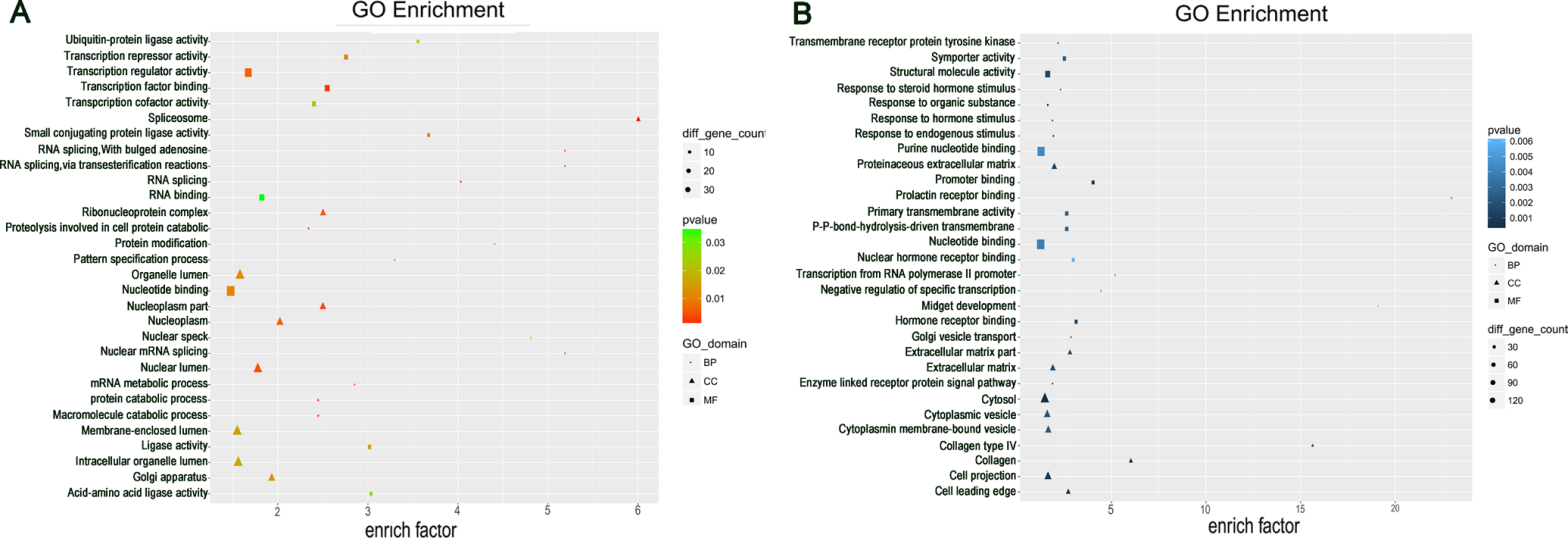
Gene Ontology (GO) analysis Go annotation of up (**A**) and down (**B**) dysregulated differentially expressed mRNAs with top thirty significant enrichment covering domains of biological processes, cellular components and molecular functions.

Studies have shown that lncRNAs could regulate the expressions of neighboring and overlapping coding genes. Therefore, Gene Ontology (GO) enrichment analysis of significantly differentially expressed mRNAs could reveal the probable role or function of related dysregulated lncRNAs. GO analysis was performed to investigate the biological processes, cellular components, and specific molecular functions of all differentially expressed mRNAs. The data showed that the biological processes associated with the upregulated mRNAs were RNA splicing and protein catabolic process. Meanwhile, majority of the genes were related to the nucleoplasm and intracellular organelle lumen in the cellular component analysis, and to transcription regulator activity in the molecular function (Figure 3A). The downregulated transcripts were mostly associated with the enzyme-linked receptor protein signaling pathway, cytosol, and nucleotide binding in the three GO classifications, respectively (Figure 3B).

### Enriched biological pathway analysis by ingenuity pathway analysis and pathway act network

**Figure 4:**
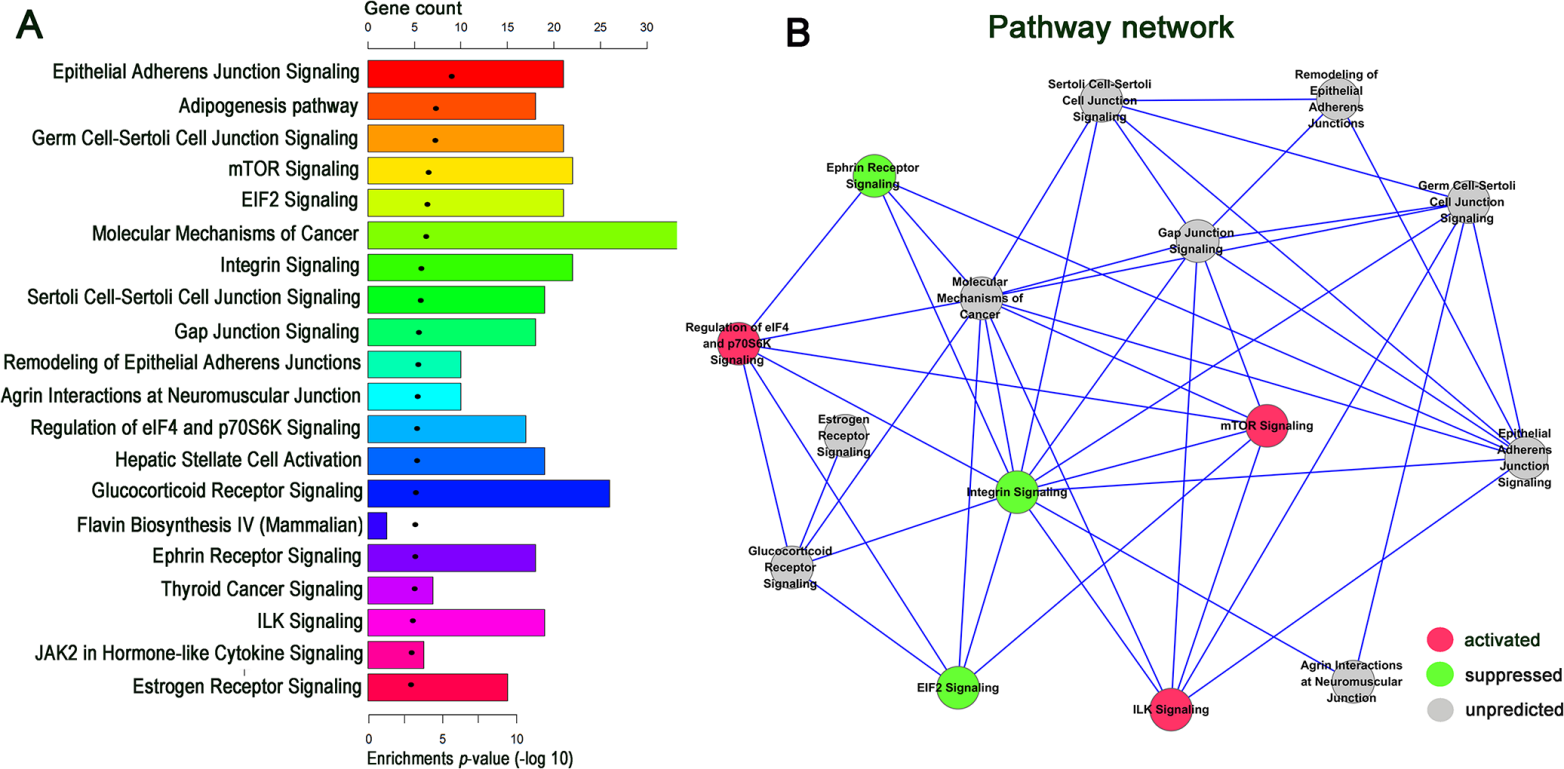
IPA enrichment analysis and pathway act network (**A**) Ingenuity Pathway Analysis of up and down regulated mRNAs enriched in the top twenty pathways according to the *p*-value. (**B**) Pathway act network according to the overlap of common differentially expressed molecules (the number of common molecules in each two pathway > 5) in the top twenty significant canonical pathways. A node represents a canonical signaling pathway. The node color is associated with pathway status. Red indicates that the signaling pathway is activated contrast to green indicates that the signaling pathway is suppressed. Grey indicates that the pathway is unpredicted.

IPA was carried out to assess which functional categories and canonical pathways were significantly dysregulated, using the 1015 differentially expressed coding genes in GA samples compared with normal tissues. Fisher's exact test identified 64 canonical pathways that were significantly [−log (P) > 1.3] enriched in tumor tissues (Supplementary Table S1). The results indicated that the mTOR, EIF2, integrin, regulation of eIF4 and p70S6K, ILK, epithelial adherens junction, germ cell-sertoli cell junction, sertoli cell-sertoli cell junction, glucocorticoid receptor, ephrin receptor, and the role of JAK2 in hormone-like cytokine signaling pathways were predicated to be among the top twenty enriched pathways according to the *P*-values (Figure 4A). These signaling pathways were involved in many physiological and pathophysiological activities; therefore, the results indicated that these pathways were likely to contribute to the tumorigenesis and development of GA.

Among the top twenty canonical pathways, the mTOR, regulation of eIF4 and p70S6K, and ILK signaling pathways were predicated to be activated, which contrasted with the predicted suppression of the EIF2, integrin, and ephrin receptor signaling pathways. IPA identified significant networks associated with the differentially expressed genes, and these networks were formed based on the number of common genes participating in any particular network. A pathway act network was constructed using the most significant top 15 pathways (the number of common genes > 5 in each two random pathways) to illustrate the key pathways in the process of GA (Figure 4B). The results suggest that the mTOR signaling pathway is the core node of the predicted pathways in the net.

### LncRNA–mRNA co-expression network

To date, the predictions of lncRNAs’ functions have been based on the annotations of the co-expressed mRNAs. We first constructed general coding–noncoding gene co-expression networks using all differentially expressed lncRNAs and mRNAs from the present study. Those lncRNAs and mRNAs that had Pearson correlation coefficients (PCCs) ≥ 0.90 were selected to construct a network using the Cytoscape program. In total, 577 lncRNAs and 100 mRNAs were included in the co-expression network. Specifically, the data showed that the co-expression network comprised 677 nodes and 2840 connections (Supplementary Figure S1). The degree parameter represents the relative pivotal role of a gene and is important to evaluate the centrality of a gene in the network analysis. The top 50 lncRNAs with the largest degree in GA were shown in Supplementary Table S2.

**Figure 5:**
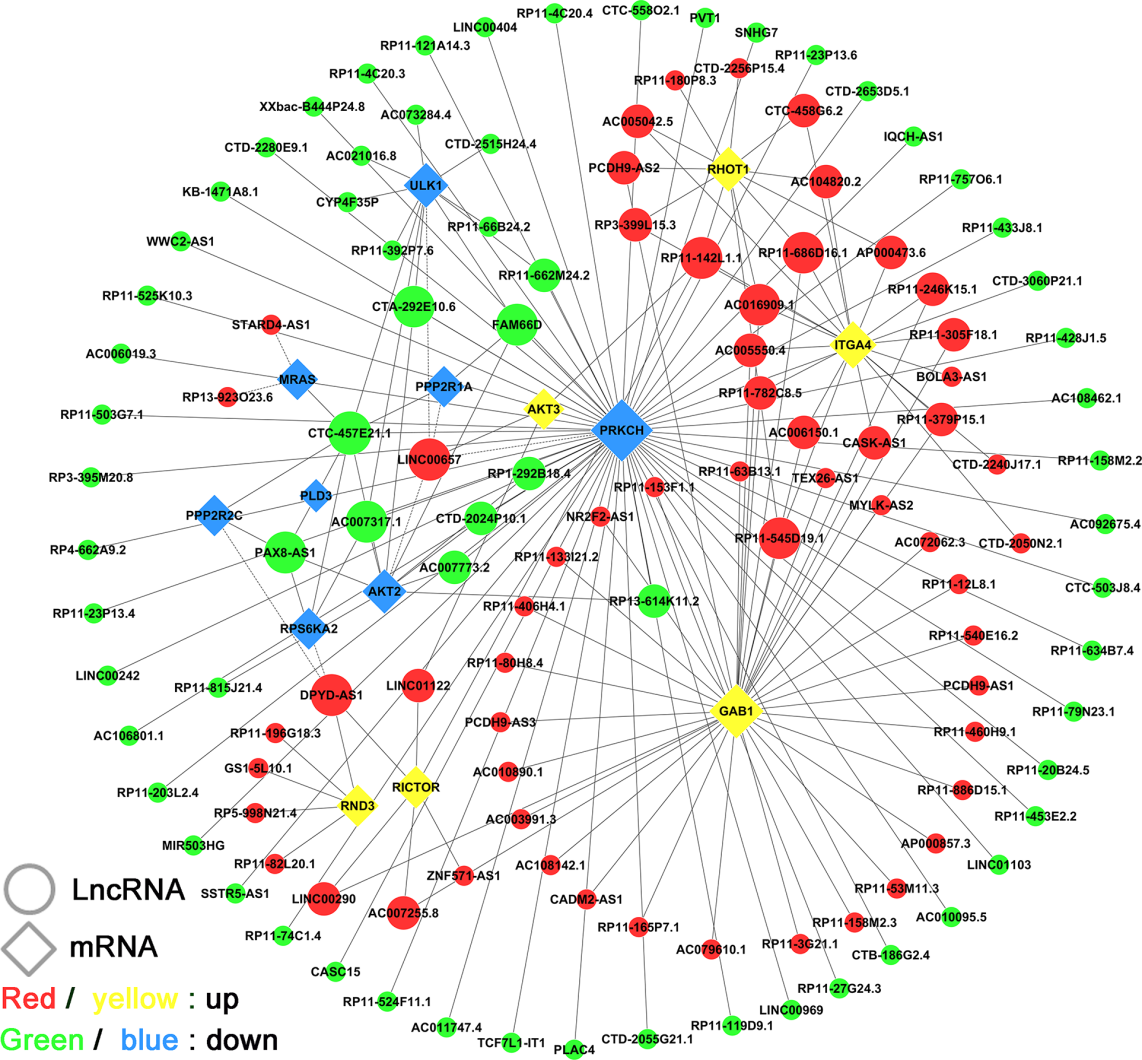
LncRNA-mRNA co-expression network in the “mTOR” pathway The network is based on Pearson correlation coefficient (the absolute value of PCC ≥ 0.80, *p*-value < 0.001). Here, 126 lncRNAs were interacting with 14 mRNAs in the meaningful “mTOR” pathway.

To identify the lncRNAs network specifically involved in the molecular pathogenesis of GA, we performed co-expression analysis using 16 key differentially expressed coding-genes (expected in IPA) of mTOR pathways. Several studies have shown that the mTOR pathway is associated with the etiology and invasiveness of GA. The sub-networks of co-expression were constructed (PCC > 0.80, *P* < 0.001), which revealed that 126 lncRNAs interacting with 14 mRNAs participated in the meaningful network pathway (Figure 5). Results demonstrated that the co-expression network comprised 140 nodes and 181 connections. The co-expression network indicated that one mRNA might correlate with 1–60 lncRNAs, and one lncRNA might correlate with one to eight mRNAs (Table 2). In particular, lncRNAs that were identified to have a documented and predominant role in the network by manual review, namely CTC-457E21.1, FAM66D, LINC00657, and PAX8-AS1, were connected to four or more differentially expressed coding genes in the mTOR pathway. These lncRNAs might play a vital regulatory role in the molecular regulation of GA.

**Table 2:** The top seven lncRNAs and mRNAs with largest degree in mTOR co-expression subnetwork

Gene symbol	LncRNAs	Degree	Gene symbol	mRNAs	Degree
	Status			Status	
CTC-457E21.1	down	8	PRKCH	down	60
LINC00657	up	5	GAB1	up	38
PAX8-AS1	down	4	ITGA4	up	19
FAM66D	down	4	RHOT1	up	12
AC007317.1	down	4	ULK1	down	12
RP11-142L1.1	up	4	AKT2	down	11
DPYD-AS1	up	4	RICTOR	up	6

### Validation of deregulated lncRNAs and mRNAs

**Figure 6:**
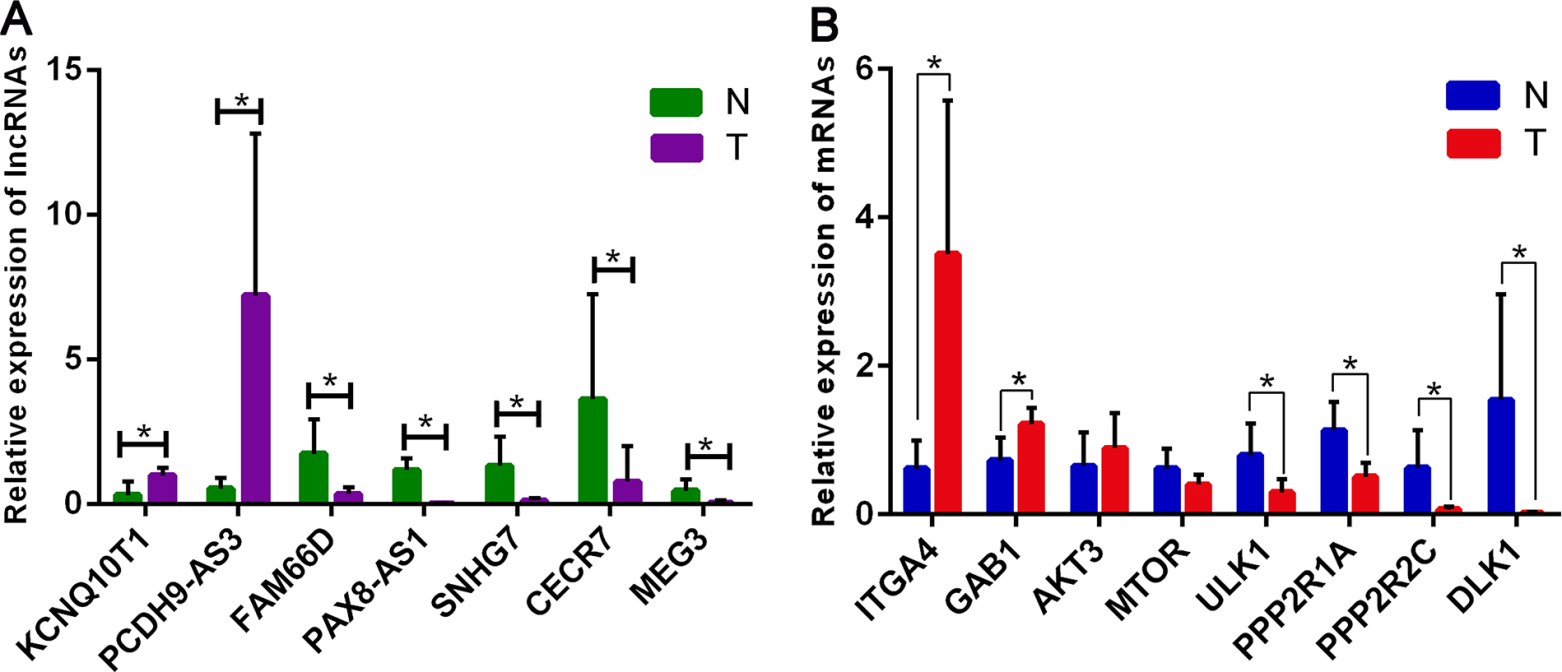
Validation of expression of significant lncRNAs and mRNAs by qRT-PCR The relative expression levels of seven lncRNAs (**A**) and eight mRNAs (**B**) are shown comparing GA tumor samples (T) with normal tissue controls (N). The relative expression level of each lncRNA and mRNA was normalized, and data displayed in histograms are presented as means ± SD, *P < 0.05.

To validate our results independently and determine the role of lncRNAs in GA, seven lncRNAs and eight mRNAs that were involved in the mTOR co-expression network were chosen for verification of the RNA-seq results by quantitative real-time PCR (qRT-PCR). Results showed that the expressions of PCDH9-AS3 and KCNQ10T1 were upregulated, whereas FAM66D, PAX8-AS1, CECR7, SNHG7, and MEG3 were downregulated in GA samples compared to in the control (Figure 6A). Meanwhile, target mRNAs *GAB1*, *AKT3*, and *ITGA4* were upregulated, and *ULK1*, *PPP2R1A*, *PPP2R2C*, and *DLK1* were downregulated in GA samples compared to in the control. In particular, the expression of *mTOR* in GA was not significantly different from that in the control (Figure 6B). Most of these results were consistent with those of RNA-seq. Thus, the qRT-PCR data verified the accuracy of the RNA-seq analysis. The findings strongly suggest that these lncRNAs and mRNAs might be related to the pathogenesis and development of GA.

## DISCUSSION

GAs derived from follicle-stimulating hormone/luteinizing hormone (FSH/LH)-producing cells are the most common adenomas among human NFPAs. The exact molecular mechanism and epigenetic features that underlie GA are unclear. Previously, lncRNAs were considered as simply transcriptional noise [17]. However, recent studies have demonstrated that lncRNAs contribute significantly to physiological processes [18–20]. Numerous studies in the last decade have shown important roles of lncRNAs with aberrant expression in human tumor pathogenesis, such as in breast cancer, colorectal cancer, and other types of malignant tumors [21–23]. However, the role and function of lncRNAs are only beginning to be understood, and their major characteristics remain to be identified. Therefore, establishing the profiles of lncRNAs that are expressed differentially in GA patients compared to in normal tissue is imperative and important.

In the present study, we investigated the lncRNAs and mRNA expression profiles of clinical samples from 11 GA patients and five normal pituitary tissue controls using RNA-seq analysis. To the best of our knowledge, this is the first study to examine differentially expressed lncRNAs in GA samples using high throughput sequence technology. We found that 839 lncRNAs were significantly differentially expressed, which included 101 upregulated and 738 downregulated lncRNAs. Approximately 40% of the deregulated lncRNAs were intergenic. Meanwhile, 1015 differentially expressed mRNAs were identified, comprising 200 upregulated and 815 downregulated mRNAs. Hierarchical clustering analysis showed that the patterns of these differentially expressed genes were obviously disparate. We also showed that the differentially expressed lncRNAs were widely distributed on each chromosome. Various degrees of abnormality of lncRNAs suggested their probable pathogenic role in GA tumorigenesis. qRT-PCR analysis verified the RNA-seq results for seven deregulated lncRNAs and eight mRNAs. Similar to previous studies, we also showed that MEG3 was significantly underexpressed in GA. Although most of the mRNA expressions were downregulated, the upregulated ones generally showed larger changes. One possible reason for this phenomenon is that the expression of most upregulated genes might be low in benign pituitary tumors, which limits the range of the functional effect. Therefore, further studies are required to investigate these inconsistencies.

Using GO and pathway analyses, we identified the enriched biological functions and potential mechanisms of the differentially expressed mRNA. GO analysis demonstrated that these genes were mainly enriched in transcription regulator activity and basic metabolic processes, including the cytosol, RNA splicing, modification-dependent macromolecule catabolic process, cell proliferation, regulation of gene-specific transcription, and protein catabolic process. Differentially expressed mRNAs were subjected to IPA to reveal the top 20 canonical pathways that probably play pivotal roles in the tumorigenesis mechanisms of GA, which included the mTOR, EIF2, integrin, regulation of eIF4 and p70S6K, ILK, and epithelial adherens junction pathways. Most of these pathways were associated with the regulation of cellular cycle, proliferation, and movement. Meanwhile, IPA predicted that three signaling pathways were activated, namely mTOR, regulation of eIF4 and p70S6K, and ILK. Overexpression of molecules of the PI3K/Akt/mTOR signaling pathway are found in many types of human cancers, and proved to activate cell growth and tumorigenesis [24–26]. Several studies have shown that the activation of PI3K/Akt/mTOR pathway is also a feature of pituitary adenomas, including NFPAs [27, 28]. Recent animal experiments have also shown that PI3K/mTOR pathway inhibition displays potential antitumor efficacy in NFPAs [27, 29]. In the present study, our results were not only found to be compatible with previous reports, but also identified the downstream pathway of eIF4 and p70S6K as being activated. *AKT* is the central mediator molecule in the PI3K/AKT/mTOR pathway and it activates crucial downstream targets, resulting in cell proliferation. Studies have demonstrated that the mRNA expression level of *AKT* is highest in NFPAs among all PA subtypes, and is considered to be associated with early recurrence of NFPAs [30, 31]. Coincidently, we also found slightly higher mRNA expression of *AKT3* in the GA samples compared with that in the normal control. However, some reports showed that the expressions of phosphorylated/total mTOR or p70S6K were not different between pituitary adenomas and controls [32, 33]. Intriguingly, these reports are consistent with our finding and these pathways should be further investigated for their potential therapeutic value. Meanwhile, our results, together with those of previous studies, suggested that other processes such as oncogene-induced senescence attenuate the changes in the downstream of PI3K/Akt/mTOR pathway in these benign tumors [34, 35].

Considering that a major function of lncRNAs is the epigenetic regulation of protein-coding genes, we could identify the putative functions of lncRNAs by detecting the expressions of their target coding genes [36–38]. The results of co-expression network analysis showed that 677 network nodes and 2840 connections were involved. This co-expression network indicated that one lncRNA could target up to 140 mRNAs and one mRNA correlate with up to 53 lncRNAs. We also built a sub-network of co-expression to reveal the molecules that probably regulated the mTOR pathway related to the pathogenesis of GAs. Results showed that 126 lncRNAs interacted with 14 mRNAs in the meaningful network pathway. Specifically, lncRNAs were identified according to the degree parameter and their documented predominant roles were reviewed manually. This approach identified CTC-457E21.1, LINC00657, PAX8-AS1, and FAM66D. LINC00657 is thought to be an oncogene; its higher expression level is correlated with poor prognosis in breast cancer, and its knockout could suppress tumor cell growth and proliferation [39]. Recent reports showed that LINC00657 was induced by hypoxia in human endothelial cells and it might stimulate in regulating cell mitosis, DNA repair, and DNA replication [40, 41]. Previous studies identified two expression quantitative trait loci (eQTLs) SNPs located in lncRNA PAX8-AS1 that might influence the expression of target oncogene PAX8 and predict a decreased risk of cervical cancer [42–44]. Additionally, KCNQ1OT1, which was one of the most upregulated lncRNAs in GA samples of our study, was also defined as an oncogene and it has been reported to have significantly higher expression levels in breast cancer and other types of malignant tumors [45, 46]. These data are consistent with our results and they support our findings that these lncRNAs are potential crucial genes and might represent new candidate molecular biomarkers in GA tumorigenesis.

In conclusion, this is the first report to demonstrate the global expression profiles of mRNAs and lncRNAs associated with GAs using high throughput sequencing technology. The dysregulated lncRNAs and mRNAs were sequenced and analyzed by GO category and IPA pathway in GA specimens. Although the regulatory roles of several lncRNAs were identified as associated with the mTOR signaling pathway, the precise regulatory mechanisms need to be further determined. The findings of this study provide a new insight into the mechanisms of GA pathogenesis and will be useful to explore candidate therapeutic targets of GA.

## MATERIALS AND METHODS

### Patients and samples

Eleven sporadic GA specimens were collected from patients who underwent transsphenoidal surgery at the Tiantan Hospital (Beijing, China). Five normal pituitary glands were used as controls, which were obtained by autopsy within 2 h of death from patients in fatal accidents. The clinical and pathological characteristics of the adenomas are described in Table 3. All tissue samples were immediately snap-frozen in liquid nitrogen and stored at −80°C. The tumor type was defined based on the pre-surgical clinical and biochemical findings, and on pathology slides. Informed consent was obtained from each patients and the study was approved by the local Ethics Committee.

**Table 3:** Clinical and pathological characteristics of patients

Patient	Sex	age (years)	Clinical features	tumor size (mm)	Immunohistochemistry
904	F	64	headache, visual defect	21 × 24 × 27	LH(+)
970	M	34	visual defect	39 × 31 × 35	FSH(+)
980	F	51	headache	17 × 33 × 24	FSH(+)
992	F	21	headache, visual defect	14 × 18 × 18	FSH(+)
994	M	45	visual defect	24 × 18 × 26	FSH(+)
1033	F	42	headache	44 × 39 × 33	Negative
1071	M	23	visual defect	23 × 29 × 25	FSH(+)
1086	F	69	visual defect	59 × 47 × 37	Negative
1134	F	47	headache, visual defect	26 × 27 × 28	Negative
1161	M	29	visual defect	14 × 18 × 19	Negative
1231	F	58	visual defect	37 × 34 × 26	Negative

* All of samples were verified by electron microscope.

### Total RNA isolation and sequencing

Total RNA was isolated from each individual sample using the TRIzol reagent (Invitrogen, USA). The purity and quantity of the total RNA were measured using Nanodrop. The integrity of RNA was assessed using the RNA Nano6000 Assay Kit of the Bionalyzer 2100 system (Agilent Technologies, CA, USA). Three micrograms of RNA per sample were used as the input material for the RNA sample preparations. First, ribosomal RNA was removed using Epicentre Ribo-zero^™^ rRNA Removal Kit (Epicentre, USA) and RnaseH; the rRNA free residue was cleaned using ethanol precipitation.

The retrieved RNA was fragmented using the Ambion Fragmentation Solution. According to the Illumina Solexa transcriptome sequencing protocol, first strand cDNA was synthesized using random hexamer primers and M-MuLV Reverse Transcriptase. The second strand cDNA synthesis was then performed using DNA Polymerase I and RNase H. In the reaction buffer, dNTPs with dTTP were replaced by dUTP. After adenylation of the 3′ ends of the DNA fragments, a NEBNext Adaptor with a hairpin loop structure was ligated to prepare for hybridization. To select cDNA fragments of the preferred 150–200 bp length, the library fragments were purified using the AMPure XP system (Beckman Coulter, Beverly, CA, USA). Finally, the products were purified (AMPure XP system) and the library quality was assessed on an Agilent Bioanalyzer 2100 system. The libraries were sequenced at the BGI–Tech Bioinformatics Institute (Shenzhen, China) on an Illumina Hiseq 2000 platform, and 90-bp paired-end reads were generated.

### Mapping and identification of differentially expressed genes

Before read mapping, clean reads were obtained by removing reads that contained adapter or poly-N, and low quality reads from raw data. At the same time, Q20, Q30, and GC contents of the clean data were calculated. All the downstream analyses were based on the high quality clean data. The clean reads were aligned to the human genome (version: GRCH37) using the HISAT2 program (V2-2.0.1) [47, 48]. We applied Stringtie (v1.3.0) and Ballgown algorithms to identify the significantly differentially expressed genes using the following criteria: FC > 2.0 or < 0.5, *P* < 0.05 and FDR < 0.1. Scatter and volcano plots were drawn using R, based on the differentially expressed gene analysis and the color was determined according to the filtering criteria. Hierarchical clustering was performed to generate an overview of the characteristics of the expression profiles, based on values of significantly differentially expressed transcripts.

### Gene ontology (GO) and ingenuity pathway analysis (IPA)

GO analysis was used to identify the biological implications of significant or representative differentially expressed genes. Those genes with significant differential expression in GA were used for GO analysis in DAVID (http://david.abcc.ncifcrf.gov/). IPA software (http://www.ingenuity.com) was used to identify the significant pathways of the differentially expressed genes. Canonical pathways and biofunctions were identified from the IPA library using the “Core Analysis” function, based on their significance to the dataset (Fisher's exact test; *P*-value < 0.05).

### Construction of pathway act network and co-expression analysis

We chose those genes enriched in the significant canonical pathways of IPA (*P* < 0.05) and used the software Cytoscape (V2.8.0) (http://www.cytoscape.org) to construct a pathway act network for graphical representations of central pathways. A co-expression network based on calculating the PCC between the differentially expressed lncRNAs and mRNAs associated with GA was constructed. To produce a visual representation, lncRNAs and mRNAs with PCCs > 0.90 were used to construct the general network using Cytoscape. In this analysis, each gene corresponded to a node and two genes linked by an edge indicated a high correlation (i.e., either positive or negative). Degree score was used to identify pivotal regulatory genes in the networks. Finally, the degree was calculated to examine the topological properties of this graph. The degree of one gene was defined as the number of directly linked neighbors.

### Quantitative real-time PCR validation

Total RNA was extracted using the TRIzol reagent (Invitrogen, USA) and then reversed transcribed using a HiFiScript gDNA Removal cDNA Synthesis Kit (CWBio, China), according to the manufacturer's instructions. Subsequently, we performed qRT-PCR using SYBR Green assays in a total reaction volume of 10 μl, including 0.5 μl of PCR Forward Primer (10 μM), 0.5 μl of PCR Reverse Primer (10 μM), 2 μl of cDNA, 5 μl of 2 × Master Mix and 2 μl of double distilled water. The protocol was initiated at 95°C for 3 min, followed by 40 cycles of 95°C (10 sec), and 60°C (60 sec). *GAPDH* was used as a reference gene. The results were harvested in three independent wells. For the quantitative analysis, the relative expression level of each gene was calculated using 2-ΔΔCt method. Student's *t*-tests were applied and a *P*-value < 0.05 was considered significant. The values were expressed as means ± SD. The primer sequences are presented in Table 4.

**Table 4:** PCR primers of lncRNAs and mRNAs used for qRT-PCR

Gene symbol	Gene type	Forward primer	Reverse primer
GAB1	mRNA	5′TGTGCGAGGGACATACAGTGAT3′	5′CCATAGCCTCACCAAGTTGACA3′
GAPDH	mRNA	5′ACAGCCTCAAGATCATCAGCAAT3′	5′GATGGCATGGACTGTGGTCAT3′
AKT3	mRNA	5′TTTCAGGGCTCTTGATAAAGGA3′	5′TCTTGCCAGTTTACTCCAGAGAA3′
PPP2R1A	mRNA	5′CCATCCTGGACAACAGCACC3′	5′CATCAGGCGAGAGACAGAACAG3′
ULK1	mRNA	5′GGTCACACGCCACATAACAGA3′	5′CCACAAGGTGAGAATAAAGCCAT3′
MTOR	mRNA	5′CAGTGCTATATTGGCTGGTGC3′	5′TCACCATGGTTTCAGTTTAGTGG3′
DLK	mRNA	5′AGACATGATCAGCAATTTGGTCC3′	5′GTCTGGACTGGTAGTAAACGGGA3′
ITGA4	mRNA	5′ATTCAGATGGCTCCACTAAAAGAT3′	5′ATTTTCTTTGGTTATGCGTGTCT3′
PPP2R2C	mRNA	5′TGGTGTGGGATACATGGTGGT3′	5′GGGTAATTTGTGAGACACCAAAG3′
PAX8-AS1	lncRNA	5′GGGGTAAAACTGTTTGAAAGTGTT3′	5′TGTTTCTCCACCCACATAGACTG3′
FAM66D	lncRNA	5′CAAGGATCTTCACACCTGGATT3′	5′CCTGTGGAAGGTCATTGTCTCAA3′
KCNQ1OT1	lncRNA	5′CAGGAGTTCAAGAATAGCATGCA3′	5′CTCAAGTGATCCTTGCCCCTT3′
PCDH9-AS3	lncRNA	5′CAGGCATCAGGATAGCTCATTT3′	5′TTATCGTTAGTCAGTCACCCTGC3′
MEG3	lncRNA	5′CTGAAGAACTGCGGATGGAAG3′	5′TAGGCATCCAGGTGATGGCT3′
CECR7	lncRNA	5′CCCCAACAAAACCCTAAACTCT3′	5′TGGAGGAAAAACTCAGTGATCG3′
SNHG7	lncRNA	5′ACCACGCCTCCCTTTTCATA3′	5′GTCTTAGGTTCCAGGCAGTTCA3′

## SUPPLEMENTARY MATERIALS FIGURE AND TABLES




