# Supplementary material for: Genome-wide analysis of differentially expressed lncRNAs and mRNAs in primary gonadotrophin adenomas by RNA-seq
Source: Oncotarget. 2016 Dec 15;8(3):4585–606. doi: 10.18632/oncotarget.13948 (PMC5354857; doi:10.18632/oncotarget.13948)
Supplement: Supplementary file 1 [file oncotarget-08-4585-s001.pdf]

Genome-wide analysis of differentially expressed lncRNAs and mRNAs in primary gonadotrophin adenomas by RNA-seq

Supplementary Materials

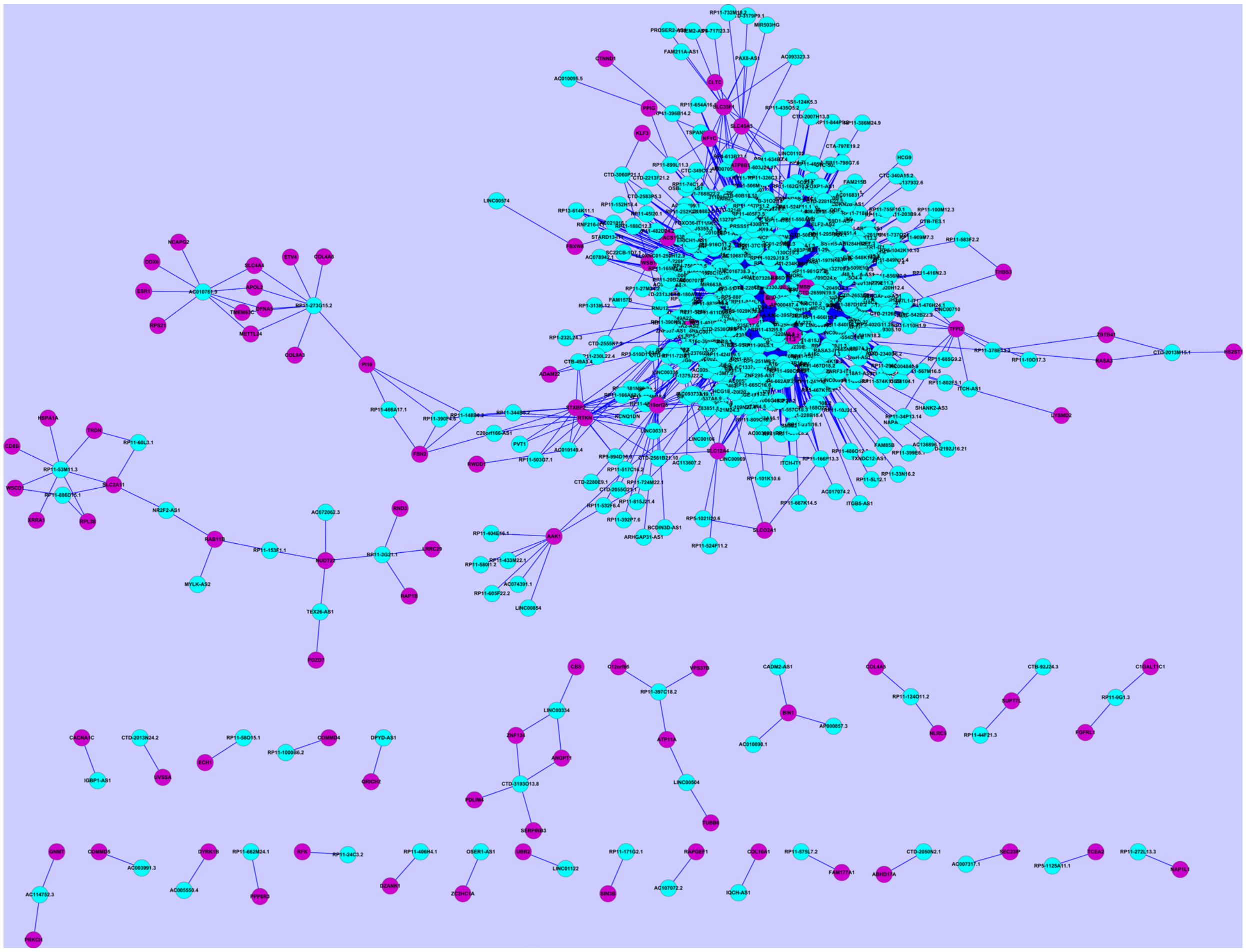

Supplementary Figure S1: Co-expression general networks in GA samples. The lines indicate a correlative relationship. The purple circles represent mRNAs and blue circles represent LncRNAs.

Supplementary Table S1: 64 significantly enriched canonical pathways identified by IPA in GA samples. See Supplementary_Table_S1

Supplementary Table S2: The top 50 lncRNAs with the largest degree in GA samples

	LncRNAs	
**Gene symbol**	**Degree**	**Log_2FC**
RP4-799P18.3	10	−4.1642
RP13-614K11.2	9	−5.8600
RP11-507J18.2	9	−1.9045
CTD-2666L21.1	9	−3.9954
AC010761.9	9	−1.7949
RP11-273G15.2	9	−2.2777
AC073284.4	8	−9.0888
AC012307.2	8	−7.7326
HCG18	8	−6.4955
AC004840.9	8	−1.8501
DBH-AS1	8	−4.7398
RP11-429J17.5	8	−5.6349
AC092155.4	8	−5.2864
SNORD3B-1	8	−5.0000
RP11-124K4.1	8	−6.6167
AC006547.13	8	−3.2474
RNU12	8	−4.5804
CYP4F35P	8	−6.1531
RP3-453P22.2	8	−2.2340
AC091729.8	8	−3.7879
RP11-109N23.4	8	−2.3524
HAR1A	8	−2.5271
CTD-2281E23.3	8	−4.4234
AC012354.6	8	−2.1126
LL09NC01-251B2.3	8	−3.4105
OR2A1-AS1	8	−3.7276
RP11-482D24.3	8	−4.2646
CTC-457E21.1	8	−2.3550
ZNF337-AS1	8	−1.8775
RP11-465N4.4	8	−1.5578
RP11-634B7.4	8	−5.1922
RP11-405F3.5	8	−3.7920
LINC00354	7	−9.9658
SSTR5-AS1	7	−9.9658
LINC00404	7	−9.9658
RP11-20I20.2	7	−9.9658
RP3-512E2.2	7	−9.9658
AC141930.2	7	−9.9658
RP11-598C10.1	7	−8.4886
AC106870.2	7	−10.6285
RP3-404K8.2	7	−12.5289
RP1-309H15.2	7	−9.3248
RP11-554D14.7	7	−8.0883
AC133785.1	7	−8.8453
RP11-554D14.6	7	−6.9484
RP13-614K11.1	7	−7.6000
RP11-168O22.1	7	−7.1760
RP11-398J5.1	7	−7.6951
RP11-532N4.2	7	−8.2682
BX322557.10	7	−7.5932
