# Supplementary material for: Genome-wide analysis of differentially expressed lncRNAs and mRNAs in primary gonadotrophin adenomas by RNA-seq
Source: Oncotarget. 2016 Dec 15;8(3):4585–606. doi: 10.18632/oncotarget.13948 (PMC5354857; doi:10.18632/oncotarget.13948)
Supplement: Supplementary file 2 [file oncotarget-08-4585-s002.docx]

**Supplementary Table S1.**

64 significantly enriched canonical pathways identified by IPA in GA samples.

| **Ingenuity Canonical Pathways** | **-log(p-value)** | **Molecules** |
| --- | --- | --- |
| Epithelial Adherens Junction Signaling | 5.04 | RAP1B,RAPGEF1,TCF4,TUBB3,AKT2,TGFBR1,MYO7A,KRAS,MYH7B,FGF1,CTNNA2,TUBA1A,TUBA8,TUBB6,MRAS,AKT3,JUP,CTNNB1,ACTG1,ACTA1,CTNND1 |
| Adipogenesis pathway | 3.98 | CCNH,HDAC4,ATG7,DGKD,SMAD3,NR2F2,SIN3B,XBP1,SAP130,SAP30,BSCL2,FGF1,HDAC5,KDM1A,DLK1,AGPAT2,KLF3,FGFRL1 |
| Germ Cell-Sertoli Cell Junction Signaling | 3.94 | TUBB3,TGFBR1,MYO7A,KRAS,GSN,CTNNA2,TUBA1A,PAK3,GAB1,RND3,TUBB6,TUBA8,RHOT1,MRAS,PAK5,MAP2K3,JUP,CTNNB1,ACTG1,ACTA1,CTNND1 |
| mTOR Signaling | 3.49 | ULK1,MAPKAP1,AKT2,PLD3,EIF4G3,RPS21,KRAS,RICTOR,RPS29,FAU,PPP2R1A,RPS16,RND3,GAB1,RHOT1,MRAS,AKT3,FIGF,PPP2R2C,PRKCH,RPS6KA2,RPS3 |
| EIF2 Signaling | 3.4 | AKT2,RPLP1,RPL34,EIF4G3,RPS21,RPL35A,KRAS,RPS29,RPL9,FAU,SHC1,RPS16,EIF1,RPL18A,GAB1,AGO3,MRAS,AKT3,RPS3,RPL31,RPL38 |
| Molecular Mechanisms of Cancer | 3.34 | RAP1B,RAPGEF1,TCF4,TGFBR1,SMAD3,KRAS,ARHGEF1,CDC25B,SHC1,CTNNA2,RHOT1,MRAS,AKT3,CTNNB1,NFKBIB,ITGA4,SMAD2,LRP5,PMAIP1,AKT2,ARHGEF12,BAX,RND3,PAK3,GAB1,PLCB3,PAK5,MAP2K3,PRKCH,CFLAR,GNAL,BIRC2,PRKAR1A,CTNND1 |
| Integrin Signaling | 3 | RAP1B,RAPGEF1,AKT2,KRAS,GSN,GIT1,SHC1,PPP1R12A,PAK3,GAB1,RND3,LIMS1,RHOT1,MRAS,AKT3,PAK5,ILKAP,CTTN,ACTG1,ITGB5,ACTA1,ITGA4 |
| Sertoli Cell-Sertoli Cell Junction Signaling | 2.96 | TUBB3,AKT2,MYO7A,KRAS,CLDN7,CTNNA2,TUBA1A,TUBB6,TUBA8,CGN,MRAS,AKT3,MAP2K3,JUP,CTNNB1,ACTG1,ACTA1,PRKAR1A,ITGA4 |
| Gap Junction Signaling | 2.83 | AKT2,TUBB3,CSNK1A1,KRAS,DRD2,TUBA1A,GAB1,TUBB6,TUBA8,MRAS,AKT3,PLCB3,PRKCH,CTNNB1,NPR2,ACTG1,ACTA1,PRKAR1A |
| Remodeling of Epithelial Adherens Junctions | 2.81 | CTNNA2,TUBB3,TUBA1A,TUBB6,TUBA8,CTNNB1,ACTG1,MAPRE3,ACTA1,CTNND1 |
| Agrin Interactions at Neuromuscular Junction | 2.76 | PAK3,UTRN,MRAS,PAK5,KRAS,ERBB2,CTTN,ACTG1,ACTA1,ITGA4 |
| Regulation of eIF4 and p70S6K Signaling | 2.73 | AKT2,EIF4G3,RPS21,KRAS,RPS29,FAU,SHC1,PPP2R1A,EIF1,RPS16,GAB1,AGO3,MRAS,AKT3,PPP2R2C,RPS3,ITGA4 |
| Hepatic Fibrosis / Hepatic Stellate Cell Activation | 2.73 | COL19A1,SMAD2,COL4A5,TGFBR1,COL4A6,SMAD3,COL4A3,COL9A3,COL10A1,BAX,MYH7B,FGF1,COL16A1,CD40,IGF1R,FIGF,ECE1,COL4A4,COL9A2 |
| Glucocorticoid Receptor Signaling | 2.64 | ARID1A,TGFBR1,PRL,SMAD3,HSPA1A/HSPA1B,POMC,KRAS,TAF13,GTF2A2,SHC1,NCOA2,MRAS,AKT3,NCOR1,NFKBIB,POLR2J2/POLR2J3,SMAD2,AKT2,CCNH,POLR2K,PCK1,SCGB1A1,GAB1,HLTF,ESR1,UBE2I |
| Flavin Biosynthesis IV (Mammalian) | 2.6 | FLAD1,RFK |
| Ephrin Receptor Signaling | 2.6 | RAP1B,RAPGEF1,AKT2,ANGPT1,EPHA1,GNG2,KRAS,EPHA3,FGF1,SHC1,PAK3,ABI1,MRAS,AKT3,FIGF,PAK5,GNAL,ITGA4 |
| Thyroid Cancer Signaling | 2.53 | SHC1,TCF4,MRAS,KRAS,RXRA,RET,CTNNB1 |
| ILK Signaling | 2.44 | AKT2,RICTOR,MYH7B,PPP2R1A,PPP1R12A,GAB1,RND3,RHOT1,LIMS1,FIGF,AKT3,KRT18,ILKAP,PPP2R2C,TMSB10/TMSB4X,CTNNB1,ACTG1,ACTA1,ITGB5 |
| Role of JAK2 in Hormone-like Cytokine Signaling | 2.34 | SHC1,PRL,SOCS2,PRLR,GH1,HLTF |
| Estrogen Receptor Signaling | 2.31 | POLR2J2/POLR2J3,CCNH,PHB2,KRAS,POLR2K,HNRNPD,PCK1,TAF13,SHC1,DDX5,NCOA2,MRAS,NCOR1,ESR1 |
| Axonal Guidance Signaling | 2.3 | RAP1B,ADAM22,ECEL1,SLIT1,KRAS,ADAMTS2,SHC1,MICAL1,TUBA8,MRAS,AKT3,FIGF,ERBB2,SHANK2,ACE,ITGA4,AKT2,TUBB3,ARHGEF12,EPHA1,GNG2,EPHA3,SLIT2,HKR1,GIT1,TUBA1A,GLIS2,TUBB6,GAB1,PAK3,PLCB3,PAK5,PRKCH,GNAL,PRKAR1A |
| Myc Mediated Apoptosis Signaling | 2.1 | SHC1,AKT2,GAB1,IGF1R,MRAS,YWHAZ,AKT3,KRAS,BAX |
| PI3K/AKT Signaling | 2.09 | SHC1,PPP2R1A,AKT2,GAB1,LIMS1,MRAS,YWHAZ,AKT3,PPP2R2C,KRAS,NFKBIB,CTNNB1,ITGA4 |
| Huntington's Disease Signaling | 2.06 | POLR2J2/POLR2J3,AKT2,HDAC4,HSPA1A/HSPA1B,GNG2,CLTC,PSME2,POLR2K,RCOR2,BAX,HDAC5,TGM2,SHC1,GAB1,CASP2,IGF1R,PLCB3,AKT3,NCOR1,PRKCH,GOSR2 |
| Tight Junction Signaling | 2.03 | AKT2,TGFBR1,MARK2,CPSF1,CASK,CLDN7,MYH7B,PPP2R1A,CGN,AKT3,PPP2R2C,GOSR2,CTNNB1,ACTG1,ACTA1,PRKAR1A |
| NF-κB Activation by Viruses | 1.97 | AKT2,GAB1,MRAS,AKT3,KRAS,PRKCH,NFKBIB,ITGB5,TNFRSF14,ITGA4 |
| 14-3-3-mediated Signaling | 1.9 | AKT2,TUBB3,YWHAZ,KRAS,BAX,TUBA1A,TUBB6,TUBA8,GAB1,MRAS,AKT3,PLCB3,PRKCH |
| RhoGDI Signaling | 1.89 | ARHGEF12,ARHGDIG,GNG2,ARHGEF1,PIP5K1B,PPP1R12A,PAK3,RND3,RHOT1,MRAS,PAK5,ACTG1,ESR1,GNAL,ACTA1,ITGA4 |
| Pyridoxal 5'-phosphate Salvage Pathway | 1.89 | PDXK,AKT2,PAK3,PRPF4B,CSNK1A1,PAK5,MAP2K3,PRKCH |
| Angiopoietin Signaling | 1.87 | AKT2,ANGPT1,GAB1,PAK3,MRAS,AKT3,PAK5,KRAS,NFKBIB |
| Myo-inositol Biosynthesis | 1.85 | ISYNA1,IMPA2 |
| Endometrial Cancer Signaling | 1.85 | CTNNA2,AKT2,GAB1,MRAS,AKT3,KRAS,ERBB2,CTNNB1 |
| phagosome maturation | 1.82 | TUBB3,LAMP2,ATP6V0B,TUBA1A,TUBB6,TUBA8,VPS37B,DYNC2H1,ATP6V1G1,GOSR2,VPS37C,ATP6V0E1 |
| Actin Cytoskeleton Signaling | 1.79 | ARHGEF12,ARHGEF1,KRAS,PIP5K1B,GSN,MYH7B,GIT1,FGF1,SHC1,DIAPH1,GAB1,PPP1R12A,PAK3,MRAS,PAK5,TMSB10/TMSB4X,ACTG1,ACTA1,ITGA4 |
| IGF-1 Signaling | 1.77 | SHC1,AKT2,GAB1,IGF1R,MRAS,YWHAZ,AKT3,SOCS2,KRAS,PRKAR1A,GRB10 |
| Telomerase Signaling | 1.71 | SHC1,AKT2,PPP2R1A,HDAC4,GAB1,ETS2,MRAS,AKT3,PPP2R2C,KRAS,HDAC5 |
| PPARα/RXRα Activation | 1.7 | SMAD2,TGFBR1,SMAD3,KRAS,GH1,CAND1,SHC1,MRAS,PLCB3,MAP2K3,NCOR1,NFKBIB,RXRA,ITGB5,PRKAR1A |
| ERK/MAPK Signaling | 1.67 | RAP1B,RAPGEF1,YWHAZ,KRAS,MKNK2,SHC1,PPP2R1A,PPP1R12A,PAK3,GAB1,ETS2,MRAS,PAK5,PPP2R2C,ESR1,PRKAR1A,ITGA4 |
| Androgen Signaling | 1.66 | POLR2J2/POLR2J3,SHC1,CCNH,NCOA2,SMAD3,GNG2,MRAS,POLR2K,PRKCH,GNAL,PRKAR1A |
| Polyamine Regulation in Colon Cancer | 1.65 | TCF4,PSME2,KRAS,CTNNB1 |
| Hereditary Breast Cancer Signaling | 1.65 | POLR2J2/POLR2J3,AKT2,GADD45B,HDAC4,ARID1A,GAB1,GADD45G,MRAS,AKT3,KRAS,POLR2K,HLTF,HDAC5 |
| PEDF Signaling | 1.61 | AKT2,TCF4,GAB1,MRAS,AKT3,KRAS,CFLAR,NFKBIB,TCF12 |
| FAK Signaling | 1.6 | AKT2,GAB1,PAK3,MRAS,AKT3,PAK5,KRAS,ACTG1,ACTA1,ITGA4 |
| Breast Cancer Regulation by Stathmin1 | 1.59 | TUBB3,ARHGEF12,GNG2,ARHGEF1,KRAS,SHC1,PPP2R1A,TUBA1A,PPP1R12A,GAB1,TUBB6,TUBA8,MRAS,PLCB3,PPP2R2C,PRKCH,PRKAR1A |
| RAR Activation | 1.58 | SMAD2,TRIM24,CCNH,AKT2,ARID1A,SMAD3,ALDH1A2,NR2F2,AKT3,NCOR1,PRKCH,SNW1,RXRA,HLTF,SCAND1,PRKAR1A |
| VEGF Signaling | 1.55 | SHC1,AKT2,EIF1,GAB1,MRAS,AKT3,FIGF,KRAS,ACTG1,ACTA1 |
| Role of NFAT in Cardiac Hypertrophy | 1.55 | AKT2,HDAC4,TGFBR1,GNG2,CSNK1A1,KRAS,HDAC5,SHC1,GAB1,IGF1R,MRAS,PLCB3,AKT3,MAP2K3,PRKCH,PRKAR1A |
| Nucleotide Excision Repair Pathway | 1.54 | POLR2J2/POLR2J3,CCNH,ERCC1,POLR2K,RAD23B |
| Cholecystokinin/Gastrin-mediated Signaling | 1.52 | SHC1,RND3,RHOT1,CREM,MRAS,PLCB3,KRAS,MAP2K3,PRKCH,GH1 |
| Regulation of Actin-based Motility by Rho | 1.52 | RND3,PAK3,PPP1R12A,RHOT1,PAK5,PIP5K1B,GSN,ACTA1,ITGA4 |
| Colanic Acid Building Blocks Biosynthesis | 1.52 | GMPPB,GMPPA,GMDS |
| Protein Ubiquitination Pathway | 1.51 | USP21,DNAJB12,UBE2A,PSMD13,UBR2,HSPA1A/HSPA1B,PSME2,THOP1,USP33,UBE3A,HSPA12A,UBE2D2,UBE2J1,HSPB11,UCHL5,UBA1,DNAJB5,UBE2D3,BIRC2,UBE2I |
| G Beta Gamma Signaling | 1.5 | SHC1,AKT2,GNG2,MRAS,AKT3,KRAS,PRKCH,GNAL,PRKAR1A |
| Chondroitin and Dermatan Biosynthesis | 1.48 | CSGALNACT1,CHPF2 |
| UDP-N-acetyl-D-glucosamine Biosynthesis II | 1.48 | UAP1,GFPT2 |
| GDP-mannose Biosynthesis | 1.48 | GMPPB,GMPPA |
| Relaxin Signaling | 1.48 | ENPP6,RAP1B,AKT2,GAB1,GNG2,MRAS,AKT3,PDE11A,NFKBIB,NPR2,APEX1,GNAL,PRKAR1A |
| Cardiac β-adrenergic Signaling | 1.43 | ENPP6,PLN,PPP2R1A,PPP1R12A,GNG2,AKAP9,MRAS,CACNA1C,PPP2R2C,PDE11A,APEX1,PRKAR1A |
| RhoA Signaling | 1.42 | ARHGEF12,RND3,PPP1R12A,EPHA1,RTKN,RHPN1,IGF1R,ARHGEF1,PIP5K1B,ACTG1,ACTA1 |
| Ceramide Signaling | 1.36 | AKT2,PPP2R1A,GAB1,SPHK2,MRAS,AKT3,PPP2R2C,KRAS,SMPD3 |
| Erythropoietin Signaling | 1.36 | SHC1,AKT2,GAB1,MRAS,AKT3,KRAS,PRKCH,NFKBIB |
| Renal Cell Carcinoma Signaling | 1.36 | RAPGEF1,AKT2,GAB1,PAK3,MRAS,AKT3,PAK5,KRAS |
| Pregnenolone Biosynthesis | 1.35 | MICAL1,MICAL3 |
| Neuroprotective Role of THOP1 in Alzheimer's Disease | 1.32 | HLA-G,ECE1,THOP1,ACE,PRKAR1A |
